# Fully-covered metal stent removal failure in case of non-malignant biliary strictures: Risk factors and resolution technique

**DOI:** 10.1055/a-2669-5801

**Published:** 2025-09-05

**Authors:** Nicolò de Pretis, Lorenzo Santaera, Luigi Martinelli, Maria Cristina Conti Bellocchi, Laura Bernardoni, Viola Fino, Adrian Miguel Pezua Sanjinez, Enrico Gasparini, Armando Gabbrielli, Luca Frulloni, Stefano Francesco Crinó

**Affiliations:** 1 19051 Medicine, University of Verona, Verona, Italy; 2 19051 Diagnostics and Public Health, University of Verona, Verona, Italy; 3 19051 The Pancreas Institute, Unit of Gastroenterology and Digestive Endoscopy, University of Verona, Verona, Italy; 4 9282 Gastroenterology and Digestive Endoscopy Unit, Santa Chiara Hospital, Provincia autonoma di Trento Azienda Provinciale per i Servizi Sanitari, Trento, Italy; 5 19034 Center for Medical Sciences (CISMed), University of Trento, Trento, Italy

**Keywords:** Pancreatobiliary (ERCP/PTCD), Strictures, ERC topics

## Abstract

**Background and study aims:**

Fully-covered-self-expandable-metal-stents (FC-SEMS) are commonly used for non-malignant biliary stricture treatment. Removal failure related to hyperplastic tissue development over the distal margin of the stent has been described but few data are available. FC-SEMS-in-FC-SEMS technique has been described in case reports to overcome FC-SEMS removal failure. Aims of this study were investigating technical success, clinical success, and safety of the FC-SEMS-in-FC-SEMS technique and identification of risk factors for FC-SEMS removal failure in patients with non-malignant distal biliary stricture.

**Patients and methods:**

Endoscopic retrograde cholangiopancreatography (ERCP) procedures performed between January 1, 2020 and May 31, 2023 for FC-SEMS removal in non-malignant distal biliary strictures were retrospectively identified and analyzed. Cases of FC-SEMS-in-FC-SEMS technique were evaluated.

**Results:**

FC-SEMS-in-FC-SEMS technique was used in 15 patients. FC-SEMS removal was achieved after a single treatment in 13 patients (86.7%). In the remaining two patients (13.3%), it was necessary to repeat treatment to achieve FC-SEMS removal, with an overall technical and clinical success of 100%. No significant adverse events were recorded. Among the 50 patients undergoing ERCP for FC-SEMS removal during the study period (median dwell stenting period of 306.5 days; Q1-Q3:160–392), failure was observed in 15 cases (30%). Previous biliary stenting and dwell stenting period > 300 days were identified as risk factors for FC-SEMS removal failure.

**Conclusions:**

FC-SMES-in-FC-SEMS technique appears to be safe and effective to overcome FC-SEMS removal failure in patients with non-malignant distal biliary strictures. Reducing dwell stenting period, especially in patients with personal history of previous biliary stenting, may reduce risk of FC-SEMS removal failure.

## Introduction

Distal biliary strictures are a common indication for endoscopic biliary stent placement. Different types of biliary stents have been developed, such as plastic stents and self-expanding metallic stents (SEMS) that might be uncovered, partially covered, and fully covered. In patients with malignant biliary strictures (mostly related to pancreatic adenocarcinoma and cholangiocarcinoma) placement of SEMS is definitive. In contrast, in patients with benign biliary strictures (arising from inflammatory conditions such as chronic pancreatitis or choledocholithiasis, autoimmune cholangiopathy or autoimmune pancreatitis, or from surgical interventions like those following liver transplantation or cholecystectomy) and long-life expectancy, stent placement is temporary 1 . Definitive data are lacking on optimal dwell stenting period of fully-covered SEMS (FC-SEMS) in non-malignant biliary strictures. Different dwell stenting periods have been proposed in several studies. In two randomized trials on biliary strictures secondary to chronic pancreatitis, proposed stent placement duration was 12 months 2 3 . In contracts, in other studies on benign biliary strictures, proposed stent retention time was between 4 and 12 months 4 5 6 . A meta-analysis of 22 studies including 1298 patients 7 showed a reduction in stricture recurrence after 6 months compared with 3 months of dwell stenting period, suggesting prolonged dwell stenting period for non-malignant biliary strictures.

In non-malignant biliary strictures, removable stents are needed, such as plastic stents or FC-SEMS, 8 which significantly reduce development of tissue ingrowth compared with uncovered and partially covered SEMS 9 10 .

Although many studies have shown a significant reduction in tissue ingrowth in FC-SEMS compared with partially covered and uncovered SEMS (Sakai et al. 2021) 11 some authors have reported rare cases of failure in FC-SEMS removal as a consequence of hyperplastic tissue ingrowth/overgrowth over the duodenal flange of the stent 2 12 13 .

In a prospective clinical study to evaluate safety and efficacy of FC-SEMS in non-malignant biliary strictures 14 , one case of removal failure of the FC-SEMS was described after dwell stenting time of 8 months due to development of mucosal hyperplasia at the margin of the FC-SEMS. However, very few data are available on FC-SEMS removability and rescue therapies in case of removal failure in patients with non-malignant biliary strictures. Removability of FC-SEMS has never been extensively studied and no risk factors for stent removal failure have been identified. Some authors reported use of FC-SEMS-in-FC-SEMS technique as a potential strategy to achieve FC-SEMS removal after removal failure due to ingrowth/overgrowth over the duodenal margin of the stent. However, to our knowledge, only three studies have investigated this technique as treatment for non-removable FC-SEMS, including seven, one, and five patients, respectively, with a clinical success rate of 100% and with no significant complications 2 13 15 . Moreover, no data are available on risk of FC-SEMS removal failure in real-life clinical practice and no risk factors for FC-SEMS removal failure have been identified.

The main aim of the present study was investigation of technical success, clinical success, and safety of the FC-SEMS-in-FC-SEMS technique in patients with non-removable FC-SEMS placed for non-malignant biliary stricture. The secondary aim was identification of risk factors for FC-SEMS removal failure in patients with non-malignant distal biliary stricture.

## Patients and methods

### Study design

This was a retrospective study of ERCP procedures performed at the Endoscopy Unit of the University of Verona between January 1, 2020 and May 31, 2023. The study was approved by the local ethics committee (1271 CESC).

### Patient selection

**Fig. 1:**
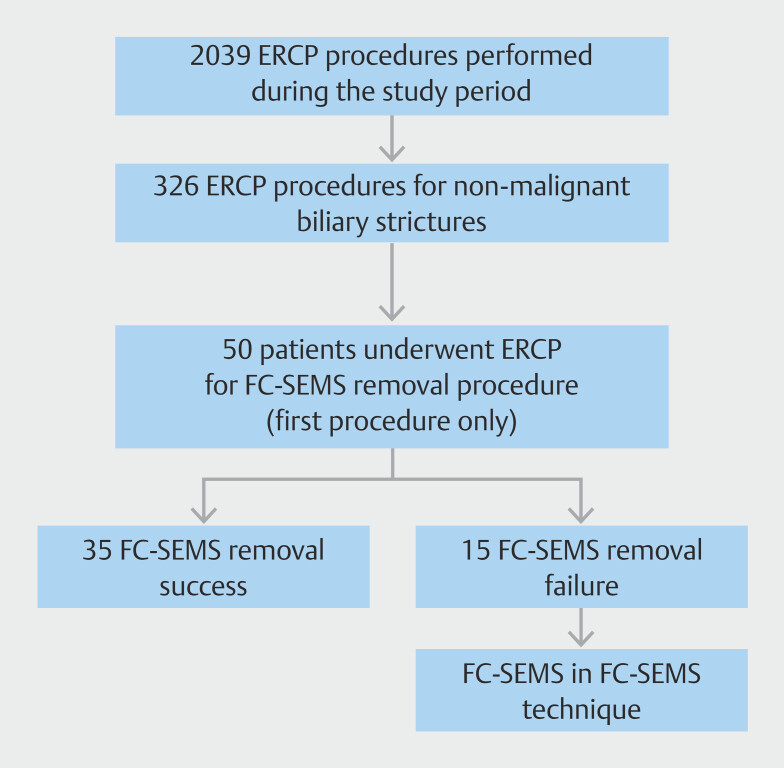
Patient flowchart. Flow diagram of patient selection and analysis. Of 2,039 ERCP procedures performed during the study period, 207 were done for FC-SEMS removal in benign biliary strictures. After applying exclusion criteria and removing repeated procedures, 50 patients were included in the final analysis. Among these, 15 experienced FC-SEMS removal failure and underwent the FC-SEMS-in-FC-SEMS technique.

Patients who underwent ERCP procedures performed between January 1, 2020 and May 31, 2023 for FC-SEMS removal in non-malignant distal biliary strictures at the Endoscopy Unit of the University of Verona were retrospectively identified from a prospectively maintained database. The patient selection process is summarized in Fig. 1 . Patients were clinically evaluated after 6 and 12 months. At our center, benign biliary strictures are usually treated with FC-SEMS over a period of 12 months. Subsequently, patients undergo an additional ERCP with FC-SEMS removal. Based on cholangiography, placement of a new FC-SEMS for an additional 12 months is considered. The same strategy is used even in patients who are not fit for surgery with biliary stricture secondary to benign or pre-malignant tumors (e.g. neuroendocrine tumors or ampullary adenoma). According to the manufacturer, FC-SEMS can be left in place for up to 12 months post-deployment. In case of FC-SEMS removal failure, the FC-SEMS-in-FC-SEMS technique is routinely applied. Written informed consent was obtained from all patients for the procedure following the Declaration of Helsinki.

Exclusion criteria were: 1) malignant biliary stricture related to pancreatic cancer, cholangiocarcinoma, neuroendocrine carcinoma, papillary cancer, lymphadenopathy, or primary duodenal cancer; 2) previous pancreatic or biliary surgery; 3).

presence of uncovered or partially covered SEMS; and 4) presence or personal history of transpapillary external drainage. current or prior transpapillary external drainage, defined as endoscopic nasobiliary drainage (ENBD) or percutaneous transhepatic biliary drainage (PTBD).

### Definitions

For the main aim of the study, procedures with FC-SEMS-in-FC-SEMS technique for FC-SEMS removal failure in patients with non-malignant distal biliary strictures were analyzed. FC-SEMS removal failure was considered as inability to completely mobilize the FC-SEMS out of the common bile duct with all the available devices (snare, basket, forceps, balloon).

Previous biliary stenting was defined as any prior endoscopic biliary stent placement, regardless of stent type (plastic or metal), performed before insertion of the FC-SEMS evaluated in the present study.

Technical success was defined as successful placement of a second FC-SEMS within the previously placed, not-removable FC-SEMS.

Clinical success was defined as successful endoscopic removal of both FC-SEMSs using the stent-in-stent technique.

### Procedure and technique

**Fig. 2:**
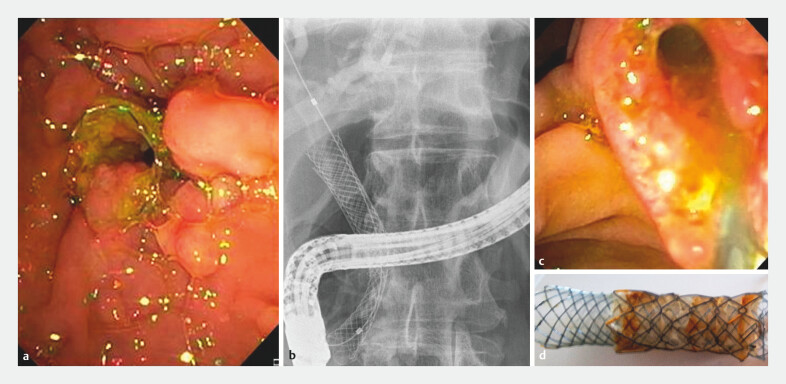
Procedure and technique. **a** Endoscopic image showing hyperplastic tissue overgrowth at the duodenal flange of the indwelling FC-SEMS, which prevents standard endoscopic removal. **b** Radiologic view of deployment of a second FC-SEMS placed coaxially inside the not-removable FC-SEMS as part of the stent-in-stent technique. **c** Endoscopic image of the papillary area after successful removal of both FC-SEMS. **d** Post-removal image showing the extracted FC-SEMSs as a single unit, with macroscopic evidence of tissue ingrowth on the outer stent.

The FC-SEMS-in-FC-SEMS technique consists of insertion of an additional FC-SEMS inside the not-removable FC-SEMS to induce necrosis of ingrowing/overgrowing hyperplastic tissue involving the duodenal margin of the stent ( Fig. 2 ). Choice of length of the additional FC-SEMS was based on length of the non-removable FC-SEMS and endoscopist preference. After 4 to 6 weeks, an additional ERCP procedure was scheduled to remove both FC-SEMS.

### Outcomes

Technical success, clinical success, and safety of the FC-SEMS-in-FC-SEMS technique were investigated. Finally, complications of the FC-SEMS in FC-SEMS technique were recorded.

To investigate the secondary aim of the study, all ERCP procedures performed during the study period for FC-SEMS removal in non-malignant distal biliary strictures were identified. If a patient underwent more than one ERCP during the study period, only the first ERCP was included in the analysis in order to comply with the assumption of independence of observations. In addition, to confirm the results, sensitivity analysis was performed to select the last available ERCP in patients who underwent more than one procedure during the study period.

At our center, distal biliary strictures secondary to non-malignant diseases are treated with FC-SEMS placement for a period of 12 months. Patients subsequently undergo an additional ERCP with FC-SEMS removal and new cholangiography to evaluate need for a new FC-SEMS insertion. Given this practice, patients were classified into three subgroups based on time between FC-SEMS placement and FC-SEMS removal attempt: 1) “early removal” if ERCP for FC-SEMS removal was attempted ≤ 300 days; 2) “standard removal” if ERCP for FC-SEMS removal was attempted between 300 and 420 days; and 3) “late removal” if ERCP for FC-SEMS removal was attempted ≥ 420 days. Demographical, clinical, endoscopic and radiological data were evaluated.

### Statistical analysis

Contingency tables were used to present frequencies and percentages of categorical variables. Given the non-normal distribution of continuous variables, these were described using medians and quartiles.

The association between duration of stent placement and patient characteristics was analyzed using non-parametric tests, specifically the Mann-Whitney and Kruskal-Wallis tests.

Variables of interest were analyzed through a logistic regression model to evaluate their impact on risk of stent removal failure. Presence of previous stenting was not included in the model due to its perfect correspondence with the outcome. Huber/White/sandwich variance-covariance matrix estimator was used to obtain robust standard errors for regression models, accounting for potential heteroscedasticity in data.

## Results

During the study period, 2039 ERCP procedures were performed. Among them, 66 (3.2%) were performed to remove a previously positioned FC-SEMS for non-malignant biliary stricture in 50 patients. Of this group, 35 patients (72%) had successful stent removal, whereas 15 patients (18%) experienced stent removal failure and required a FC-SEMS-in-FC-SEMS technique. Of patients who underwent more than one ERCP for FC-SEMS removal during the study period, only the first was considered for the analysis to comply with the assumption of independence of the observations. In detail, 36 patients underwent a single ERCP, whereas 12 and 2 patients underwent two and three ERCP procedures, respectively. All patients underwent biliary sphincterotomy prior to biliary stent insertion, except for one patient who presented with an ampulloma.

Forty patients (80%) had previous biliary stenting. Of them, 24 had previous biliary stenting with FC-SEMS and 16 with biliary plastic stents.

### FC-SEMS-in-FC-SEMS technique

Fluoroscopic view showing the placement of a fully covered self-expandable metal stent (FC-SEMS) within another previously inserted FC-SEMS (stent-in-stent technique).Video 1

During the study period, the FC-SEMS-in-FC-SEMS technique was applied in 15 patients with non-malignant distal biliary strictures to overcome FC-SEMS removal failure. The main clinical features of these patients are reported in Table 1 .

**Table:** **Table 1** Comparison of patients with endoscopic FC-SEMS removal success and failure.

	Removal success (n = 35)	Removal failure (n = 15)	*P* value
**Male** no. (%)	30 (85.7%)	13 (86.6%)	1.000
**Age** (yr) median (Q1-Q3)	64 (54–69)	59 (57–76)	0.937
**Biliary stricture**			0.076
chronic pancreatitis no. (%)	12 (34.3%)	8 (53.6%)	
AIP no. (%)	7 (20.0%)	1 (6.6%)	
NET no. (%)	3 (8.6%)	1 (6.6%)	
post-necrotic AP no. (%)	1 (2.9%)	3 (20.0%)	
ampulloma no. (%)	1 (2.9%)	1 (6.6%)	
other causes no. (%)	11 (31.4%)	1 (6.6%)	
**FC-SEMS length**			0.733
60 mm no. (%)	9 (25.7%)	5 (33.3 %)	
40 mm no. (%)	26 (74.3%)	10 (66.7%)	
**Previous biliary stenting history** no. (%)	25 (71.4)	15 (100.0)	**0.022**
**Dwell stenting period (days)** median (Q1-Q3)			
early removal (n = 25)	149 (84–252)	247 (157–287)	0.270
standard removal (n = 17)	384 (349–395)	377.5 (371.5–387.5)	0.963
late removal (n = 8)	487 (459–542.5)	1007.5 (747–1335)	0.029
All FC-SEMSs had a 10-mm diameter. Early removal indicates ERCP for FC-SEMS removal attempted ≤ 300 days after FC-SEMS placement. Standard removal indicates ERCP for FC-SEMS removal attempted between 300 and 420 days after FC-SEMS placement. Late removal indicates ERCP for FC-SEMS removal attempted ≥ 420 days after FC-SEMS placement. AIP, autoimmune pancreatitis; AP is acute pancreatitis; ERCP, endoscopic retrograde cholangiopancreatography; FC-SEMS, fully-covered-self expandable metal stent; NET, neuroendocrine tumor.			

Technical success of placement of the second FC-SEMS inside the previous one was 100% ( Video 1 ). The additional ERCP procedure for removal of both FC-SEMSs was performed after a median time of 53 days (Q1-Q3 39–65) after placement of the second FC-SEMS.

Endoscopic removal of both FC-SEMSs was achieved in 13 patients after a single treatment. In two patients, FC-SEMS was still not-removable and the FC-SEMS-in-FC-SEMS technique was repeated, achieving subsequent FC-SEMS removal in one patient. In the last patient, an additional third FC-SEMS-in-FC-SEMS period was needed for FC-SEMS removal.

The overall success rate for the FC-SEMS-in-FC-SEMS technique for overcoming failed FC-SEMS removal was 100%. No significant adverse events (AEs) were recorded, but in one patient, spontaneous migration of both FC-SEMSs was observed.

During follow-up 6 and 12 months after stent removal, no significant late AEs were reported. Only one patient died 11 months after the ERCP procedure due to heart failure at age 83.

### FC-SEMS removal failure in non-malignant distal biliary strictures

Fifty patients underwent ERCP for FC-SEMS removal during the study period. Forty-three patients were male (86%) and seven female (14%), with a median age of 62.5 years (Q1-Q3: 55–71). Chronic pancreatitis was the most common cause of biliary stricture in this population (40%), followed by autoimmune pancreatitis (16%), post-severe acute pancreatitis stricture (8%), neuroendocrine tumor (8%), ampulloma (4%) and other (24%). Median dwell stenting period before the removal attempt was 306.5 days (Q1-Q3: 160–392) and the previously placed FC-SEMS was 10 × 40 mm in 36 patients (72%) and 10 × 60 mm in 14 patients (28%). Finally, 10 patients (20%) underwent ERCP to remove the first biliary stent ever placed, whereas 40 patients (80%) had a previous biliary stenting history.

Endoscopic FC-SEMS removal was achieved in 35 patients (70%) and failed in 15 patients (30%) because of presence of hyperplastic tissue involving the duodenal flange of the stent. No differences were observed between these two groups in terms of sex, age, origin of the biliary stricture, or FC-SEMS length. However, in patients with FC-SEMS removal failure, median dwell stenting period was significantly longer (378 days Q1-Q3: 343–716 vs. 256 days Q1-Q3 117–384; *P* = 0.004). Moreover, all 15 patients with FC-SEMS removal failure had a personal history of previous biliary stenting, compared with 25 of 35 patients (71.4%) in whom FC-SEMS removal was successful ( *P* = 0.02) ( Table 1 ).

No significant differences in dwell stenting period were detected based on sex, age, FC-SEMS length, or etiology of the biliary stricture ( **Supplementary Materials** ). Results from both unadjusted logistic models showed that patients undergoing ERCP for FC-SEMS removal between 301 and 420 days after placement (standard removal) and > 420 day after placement (late removal) had a significantly increased risk of FC-SEMS removal failure compared with patients undergoing ERCP for FC-SEMS removal < 300 days (early removal) ( Table 2 ).

**Table:** **Table 2** Adjusted odds ratio analysis for FC-SEMS removal failure.

	Unadjusted OR (95% CI)	*P* value
Sex		
Female	1*	
Male	1.08 (0.18–6.44)	0.930
Age	1.01 (0.96–1.06)	0.733
FC-SEMS length		
40 mm	1*	
60 mm	1.44 (0.38–5.45)	0.587
Dwell period		
≤ 300 days	1*	
301–420 days	6.52 (1.38- 30.79)	0.018
> 420 days	7.33 (1.15–46.92)	0.035
*Reference value. FC-SEMS, fully-covered self-expanding metal stent; OR, odds ratio.		

No complications were observed in the 35 patients with FC-SEMS removal, but one case of mild acute pancreatitis was recorded among the 15 patients with FC-SEMS removal failure, with an overall risk of acute pancreatitis of 2%. No spontaneous stent migrations were observed.

Sensitivity analysis selecting the last available ERCP in patients who underwent more than one procedure in the study period confirmed the above reported results ( **Supplementary Materials** ).

## Discussion

Removal of biliary FC-SEMS can fail due to hyperplastic ingrowth/overgrowth. In patients with biliary strictures, FC-SEMS are routinely used to maintain biliary patency and to improve biliary stricture over time. Particularly in patients with non-neoplastic biliary strictures, removal of FC-SEMS needs to be achieved considering the long life expectancy and risk of cholangitis. However, very few data are available on risk of FC-SEMS removal failure or technical and clinical success of the FC-SEMS-in-FC-SEMS technique for non-removable FC-SEMS in biliary strictures. The hypothesis is that insertion of a FC-SEMS inside the non-removable FC-SEMS promotes development of ischemic necrosis of hyperplastic tissue involving the duodenal margin of the stent.

Tringali and colleagues were the first to publish a series of five patients with different etiologies of biliary stricture (2 post-cholecystectomy, 2 following liver transplantation and 1 related to chronic pancreatitis), undergoing ERCP with FC-SEMS-in-FC-SEMS for failure of FC-SEMS removal. The authors reported 100% FC-SEMS removal. Moreover, in a study involving 80 patients with distal biliary strictures secondary to chronic pancreatitis, Ramchandani and colleagues reported seven cases of FC-SEMS-in-FC-SEMS technique for FC-SEMS removal failure with technical and clinical success of 100%. The present paper represents the largest series of patients treated with this technique. We confirmed that the technique is easy, with technical success of 100%, and effective with clinical success of 86.7% after a single treatment, with a mean dwell period of the second FC-SEMS of 53 days. In addition, we observed that prolonging treatment is a possible and effective strategy if the FC-SEMS still appears to be unremovable, with overall clinical success of 100%.

As in the studies of Tringali and Ramchandani, we did not observe significant AEs but one patient had spontaneous migration of both FC-SEMSs. Therefore, in patients with known or suspected bowel strictures (e.g. Crohn disease or post-surgical adherences), this strategy should be considered with caution 16 . Otherwise, the FC-SEMS-in-FC-SEMS technique appears to be a safe and easy strategy for non-removable FC-SEMS in patients with distal biliary stricture.

Moreover, prevalence of FC-SEMS removal failure was investigated. To our knowledge, no data are available on this topic. Although FC-SEMS have significantly reduced the problem of tissue ingrowth compared with uncovered or partially covered SEMS, involvement of the duodenal margin by hyperplastic tissue may hinder removal of FC-SEMS. In the present paper, FC-SEMS removal failure was observed in 30% of cases, suggesting that this complication might be more frequent than previously thought. Ramchandani and colleagues published a study including 80 patients with distal biliary strictures secondary to chronic pancreatitis, treated with FC-SEMS for 12 months. The authors reported FC-SEMS removal failure in seven cases (8.7%). This difference might be explained by the different clinical settings. The Ramchandani study was a randomized controlled trial with strict inclusion criteria, limited to patients with chronic pancreatitis, and precise timing of endoscopic procedures. The present study, according to the retrospective design, included patients with different dwell stenting periods and different causes of biliary strictures. Moreover, the present study period included the COVID-19 pandemic, during which some scheduled endoscopic procedures were postponed, explaining some prolonged FC-SEMS dwell periods.

Patients were classified based on dwell stenting period as “early removal,” “standard removal,” or “late removal”. Patients undergoing “early removal” (within 300 days from FC-SEMS placement) had significantly lower risk of FC-SEMS removal failure. A possible explanation is that prolonging the dwell period beyond 300 days may promote hyperplastic tissue development over the duodenal flange of the FC-SEMS. This might be further confirmed by the observation that FC-SEMS removal failure was observed only in patients with a previous history of biliary stenting. These factors may promote a chronic inflammatory response to the biliary stent, considered a foreign body, with subsequent hyperplastic tissue development at the duodenal stent margin, complicating removal.

Removal failure was not found in any patients who underwent ERCP for removal of the first FC-SEMS ever positioned.

Moreover, no factors other than dwell stenting period and history of previous biliary stenting were associated with FC-SEMS removal failure. Further studies could analyze the role of other ERCP-specific procedural factors, such as length of the stent portion protruding from the papilla.

Interestingly, one patient developed mild post-ERCP pancreatitis after a failed FC-SEMS removal attempt. This may suggest that persevering in difficult stent removal may lead to AEs, similar to the experience of Tringali and colleagues, who reported one case of mild acute pancreatitis and one of self-limited hemobilia as a result of the attempted FC-SEMS removal.

Therefore, in case of difficult FC-SEMS removal related to hyperplastic tissue on the duodenal margin, risk of acute pancreatitis should be considered, probably based on traction on the papillary area. In this case, interruption of removal attempts and the FC-SEMS-in-FC-SEMS technique should be considered as a safe alternative strategy.

The retrospective nature of the present study represents a main limitation in terms of causality, with potential selection bias, partially compensated for by presence of a prospectively maintained database. Moreover, despite being the largest series of patients treated with the FC-SEMS-in-FC-SEMS technique to our knowledge, the overall sample size is still limited. Nevertheless, the collected data provide preliminary insights for larger prospective studies. Moreover, because this analysis was conducted in a single center, the results may not be immediately generalizable to other clinical settings. Multicenter studies are needed to confirm the reproducibility of these findings.

## Conclusions

In conclusion, we confirmed that the FC-SEMS-in-FC-SEMS technique appears to be a valid and safe therapeutic option in case of failure of removal of FC-SEMS in patients with non-malignant distal biliary strictures. Moreover, failed removal of FC-SEMS appears to be a frequent complication during non-cancer-related distal biliary stricture treatment. Previous biliary stenting and dwell period longer than 300 days appear to be risk factors for FC-SEMS removal failure. Therefore, the dwell stenting period should probably not exceed 300 days, especially in patients with a previous history of biliary stenting.

## Supplementary Material

Supplementary Material Zusatzmaterial
