# Supplementary material for: Fully-covered metal stent removal failure in case of non-malignant biliary strictures: Risk factors and resolution technique
Source: Endosc Int Open. 2025 Sep 5;13:a26695801. doi: 10.1055/a-2669-5801 (PMC12417772; doi:10.1055/a-2669-5801)
Supplement: Supplementary file 2 — Supplementary Material [file 10-1055-a-2669-5801_26732242.pdf]

Supplementary Table 1 Comparison of dwell stenting period between different subgroups.

	Dwell stenting period (days) median (Q1-Q3)	*P*
**Sex**		0.338
**Male**	343.0 (160.0-395.0)	
**Female**	210.0 (110.0-377.0)	
**Age**		0.324
**< 55 years**	345.5 (113.5-395-5)	
**55-64 years**	376.5 (292.5-400.5)	
**65-74 years**	228.5 (110.0-359.0)	
**> 75 years**	249.5 (161.0-380.5)	
**Biliary stricture**		0.079
**Chronic pancreatitis**	353.5 (264.5-393.5)	
**AIP**	139.5 (99.0-229.0)	
**NET**	500.5 (206.0-655.5)	
**Post-necrotic AP**	389.5 (359.0-447.0)	
**Ampulloma**	722.5 (12.0-1433.0)	
**Other causes**	254.0 (179.5-363.0)	
**FC-SEMS length**		0.484
**60 mm**	299.5 (81.0-392.0)	
**40 mm**	306.5 (188.0-392.5)	
**FC-SEMS removal**		0.004
**Success**	256.0 (117.0-384.0)	
**Failure**	378.0 (343.0-716.0)	

AIP is autoimmune pancreatitis; AP is acute pancreatitis; FC-SEMS, fully-covered-self expandable metal stent; NET is neuroendocrine tumor.

Supplementary Table 2 Sensitivity analysis selecting the last available ERCP in patients underwent more than 1 procedure in the study period.

	**Unadjusted OR (CI 95%)**	***P* value**
**Sex**		
Female	1*	
Male	1.17 (0.12-11.52)	0.895
**Age**	1.03 (0.97-1.09)	0.350
**FC-SEMS length**		
40 mm	1*	
60 mm	0.40 (0.43-3.74)	0.423
**Dwell period**		
≤ 300 days	1*	
301-420 days	1.53 (0.19- 12.35)	0.688
> 420 days	11.50 (1.52-86.90)	**0.018**

*Reference value.
CI, confidence inbterval; ERCP, endoscopic retrograde cholangiopancreatography; FC-SEMS, fully-covered self-expandable metal stent; OR, odds ratio.
